# Good animal welfare makes economic sense: potential of pig abattoir meat inspection as a welfare surveillance tool

**DOI:** 10.1186/2046-0481-65-11

**Published:** 2012-06-27

**Authors:** Sarah Harley, Simon More, Laura Boyle, Niamh O’ Connell, Alison Hanlon

**Affiliations:** 1 Wellcome Trust Research Scholar at UCD Veterinary Sciences Centre, University College Dublin, Belfield, Dublin 4, Ireland; 2 Professor of Veterinary Epidemiology and Risk Analysis, UCD Centre for Epidemiology and Risk Analysis, Veterinary Sciences Centre, University College Dublin, Belfield, Dublin 4, Ireland; 3 Senior Research Officer for Animal Behaviour and Welfare in Pig Development Department, Animal & Grassland Research & Innovation Centre, Moorepark, Fermoy, Co, Cork, Ireland; 4 Lecturer School of Biological Sciences, Medical Biology Centre, Queens University Belfast, 97 Lisburn Road, Belfast, BT9 7BL, Northern Ireland; 5 Senior Lecturer School of Veterinary Medicine, UCD Veterinary Sciences Centre, University College Dublin, Belfield, Dublin 4, Ireland

**Keywords:** Animal welfare, Surveillance, Meat inspection, Data recording, Pigs

## Abstract

During abattoir meat inspection pig carcasses are partially or fully condemned upon detection of disease that poses a risk to public health or welfare conditions that cause animal suffering e.g. fractures. This incurs direct financial losses to producers and processors. Other health and welfare-related conditions may not result in condemnation but can necessitate ‘trimming’ of the carcass e.g. bruising, and result in financial losses to the processor. Since animal health is a component of animal welfare these represent a clear link between suboptimal pig welfare and financial losses to the pig industry.

Meat inspection data can be used to inform herd health programmes, thereby reducing the risk of injury and disease and improving production efficiency. Furthermore, meat inspection has the potential to contribute to surveillance of animal welfare. Such data could contribute to reduced losses to producers and processors through lower rates of carcass condemnations, trimming and downgrading in conjunction with higher pig welfare standards on farm. Currently meat inspection data are under-utilised in the EU, even as a means of informing herd health programmes. This includes the island of Ireland but particularly the Republic.

This review describes the current situation with regard to meat inspection regulation, method, data capture and utilisation across the EU, with special reference to the island of Ireland. It also describes the financial losses arising from poor animal welfare (and health) on farms. This review seeks to contribute to efforts to evaluate the role of meat inspection as a surveillance tool for animal welfare on-farm, using pigs as a case example.

## Background

There is no universal definition of meat inspection (MI) [1]. Ante- and post-mortem MI were originally introduced to provide assurance that animal carcasses were fit for human consumption [2]. It is now recognized that such inspections also play an integral role in assessment of animal health and zoo-sanitary status, as well as detection of certain welfare conditions [3,4]. EFSA reported three primary purposes of MI: public health, animal health and meat quality [1]. References to MI in this paper will observe this EFSA definition.

During abattoir MI, carcasses with pathological lesions resulting from disease or injury are partially or fully rejected on grounds of public health or consumer acceptability. Such lesions often reflect the standard of the housing and husbandry of the animal during the production period [5,6]. Hence there is good potential to develop MI as a surveillance tool for animal welfare (AW) on farm [7,8].

This review examines the process of MI of pigs in the EU and in particular on the island of Ireland, including reporting mechanisms and economic costs to the farmer and the processor, to assess the potential of MI for surveillance of pig welfare at farm level.

## Legislative provisions for abattoir MI in the EU with special reference to the island of Ireland

### Outline of meat inspection practices

MI began in the late 1800s when transmission of zoonotic infectious disease through consumption of animal products was first recognised [2]. At that time the objective was to identify and discard carcasses infected with major zoonotic pathogens such as *Mycobacteria spp.* and parasitic infections to reduce public health risks associated with consumption of animal products. Since then MI has become highly controlled by numerous EU regulations and directives [3,9]. An overview of EU legislation controlling activities in pig meat plants is provided in Table 1.

**Table 1:** An overview of EU legislation controlling activities in pigmeat plants

**EU legislation**	**Areas covered by EU legislation**					
	**Food safety**	**Notifiable disease**	**Animal welfare**	**Quality**	**Trade**	**EU legislation**
Control of micro-organisms and implementing rules for hygeine measures	X			X	X	REGULATION (EC) No 2073/2005 of 15 November 2005 on microbiological criteria for foodstuffs, Annex 1, Chapter 2.1
Compliance with feed and food law, animal health and animal welfare rules	X	X	X		X	Regulation (EC) 882/2004 of 29 April 2004 on official controls to ensure the verification of compliance with feed and food law, animal health and AW rules
Monitoring for residues of prohibited substances	X			X	X	Directive 96/23/EC of 29 April 1996 on measures to monitor certain substances and residues thereof in live animals and animal products
Surveillance for Trichinella	X	X			X	Regulation (EC) No 2075/2005 of 5 December 2005 laying down specific rules on official controls for Trichinella in meat Regulation (EC) No 854/2004 of 29 April 2004 laying down specific rules for the organization of official controls on products of animal origin intended for human consumption
Audits of good hygiene practices (Sanitation Standard Operating Procedures (SSOP))	X			X	X	Regulation (EC) No 854/2004 of 29 April 2004 laying down specific rules for the organization of official controls on products of animal origin intended for human consumption, Article 4, 4
Rules for official controls regarding checks on:						Regulation (EC) No 854/2004 of 29 April 2004 laying down specific rules for the organization of official controls on products of animal origin intended for human consumption
Surface of skin and fractured bones		X	X	X		
Exsanguinations			X	X		
Emaciation			X	X		
Sexual odour				X		
Faecal contamination	X			X	X	
Microbiological content of foodstuffs	X				X	Regulation (EC) No. 2073/2005 of 15 November 2005 on microbiological criteria for foodstuffs
General hygiene requirements for reducing risk of pathogens present on the meat	X	X			X	Regulations (EC) No. 852/2004 and 853/2004 of 29 April 2004 laying down specific hygiene rules for food of animal origin
Monitoring of zoonoses	X				X	Directive 2003/99/EC of 17 November 2003 on the monitoring of zoonoses and zoonotic agents
Control of Salmonella	X				X	Regulation (EC) No. 2160/2003 of 17 November 2003 on the control of salmonella and other specified food-borne zoonotic agents

Sources: [3,10].

In April 2004, Regulation (EC) No 854/2004 of the European Parliament and of the Council of 29 April 2004 was introduced as part of the ‘EU Hygeine Package’ laying down specific rules for the organisation of official controls on products of animal origin intended for human consumption [11]. In this regulation the requirements and purposes of each stage of the inspection process as well as the responsibilities of the various participants are provided (see Table 2 for main provisions).

**Table 2:** Provision of EC Regulation 854/2004 on official controls of food of animal origin

**Stage**	**Requirement**	**Purpose**
Ante-mortem inspection	Must occur within 24 hours of animals arriving at slaughterhouse	Early identification of notifiable diseases
	Slaughter must occur within 24 hours of ante-mortem inspection occurring	Detection of conditions that cannot be detected at post-mortem inspection
		Detection of welfare issues
Post-mortem inspection	Occurs immediately following stunning, bleeding, scalding and evisceration of pigs	Preventing meat that is unfit for human consumption from entering the food chain
		Detection of disease lesions that pose a risk to public health, animal health or AW
Responsibilities of Official Veterinarians	Ante-mortem or Food Chain Information inspection	Preventing meat unfit for human consumption from entering the food chain
	Final post-mortem MI of carcasses at least once daily	Ensuring high standards of AW are maintained before and during slaughter
	Adherence to AW legal standards	
	Removal, treatment and disposal of Specific Risk Material and other animal by-products	
	Trichinella and prohibited substances residue testing	
Responsibilities of Veterinary Inspectors	Ante-mortem or Food Chain Information inspection, post-mortem inspection	Detection of disease lesions that pose a risk to public health, animal health or AW
Responsibilities Of Official Auxiliaries	Preliminary ante-mortem inspection to identify ‘suspect’ animals for Official Veterinarian to inspect.	Preventing meat unfit for human consumption from entering the food chain
	Post-mortem inspection	
Responsibilities of Food Business Operators	Ensuring all necessary Food Chain Information has been provided by the producer	Ensure animals are fit for slaughter

Sources: [3,12,13].

The regulation specifies disease lesions that must be identified during abattoir MI [1,12]. Detection of one or more of these conditions at ante-mortem inspection will result in condemnation of the entire carcass. At post-mortem inspection partial or full condemnation may occur [12,13]; pathological lesions localised to one anatomical region of the carcass causes partial condemnation whereas generalised conditions result in condemnation of the entire carcass [12,13].

Traditionally MI has comprised visual, palpatory and incisory techniques originally outlined by Von Ostertag in 1892 [1,2,14]. However, the emergence of various zoonotic microbiological pathogens that cannot be detected by routine MI has prompted consideration of reform of MI procedures [1-3]. Studies evaluating the efficacy of purely visual MI concluded that any decrease in lesion detection sensitivity would present a negligible increase in risk to public health [1,15,16], particularly in the context of a significant reduction in the risk of cross-contamination with microbiological pathogens (e.g. *Salmonella spp.*) [16]. Hence proposed changes include reducing routine post-mortem MI to only include visual techniques [17,18].

### Variation in implementation of EU legislation across EU member states

The EU Hygiene package allows flexibility in the way regulations are implemented by member states, and as a result some variation exists. Additionally, differences in compliance between member states have an impact; combined findings from EFSA and FVO indicate that though in principle most EU member states conduct traditional MI according to Regulation (EC) 854/2004, ante- and post-mortem MI is deficient with regard to specific areas of the regulations in a number of member states [3].

One of the main areas where differences exist across the EU is in the structure and training requirements of the veterinary public health workforce (Table 3). Some member states (e.g. Italy, Greece) require significant post-graduate veterinary training for routine MI [19] whilst others (e.g. UK, NI) employ non-veterinary workers known as Official Auxiliaries. In the case of the latter, the proportion of Veterinary Inspectors to Official Auxiliaries in the veterinary public health workforce is highly variable (e.g. Austria 20:1, Germany 1.2:1) [19].

**Table 3:** A summary of the EU requirements and examples of member state organization of training for meat inspection professionals

**Country**	**Non-veterinary**		**Veterinary post-graduate**		**Meat inspection professionals**
	**Type of training/assessment**	**Duration**	**Type of training/assessment**	**Duration**	
**EU regulation**	**Theoretical training**	**500 hours**	**Probationary training in food businesses**	**200 hours**	
	**Practical training**	**400 hours**			
	**Mandatory assessment**		**Assessment at discretion of competent authority**		
Denmark	Formal training at Danish Meat Hygiene College	6 months			Official Auxiliaries
					Official Veterinarians
France	Theoretical training	54 weeks			Official Auxiliaries
	Practical training and assessment	35 weeks			Official Veterinarians
Finland	Theoretical	Short			Official Auxiliaries
					Official Veterinarians
Ireland: Republic	National certificate level/equivalent in agricultural studies		Theoretical and practical training under supervision of Technical Agricultural Officers And Official Veterinarians	2 weeks	Technical Agricultural Officers
	Theoretical and practical training.	24 hours			Veterinary Inspectors
	Modular training:- Practical- Theory- Assessment	24 hours			Temporary Veterinary Inspectors
Ireland: Northern	Theoretical		Theoretical and written assessment	5 days	Official Auxiliaries
			Practical experience AW course at Bristol university	1 week	Official Veterinarians
Italy	N/A		Practical experience and written assessment	45 days	Official Veterinarians
			Post-graduate courses in VPH:		Veterinary Inspectors
			Diplomas	1 year	
			Special Certificates	2 years	
Luxembourg	N/A		Experience in ‘food facilities’ practical examination		
Netherlands			Course to become full-time VPH workers in Meat Hygiene Service	12 weeks	Official Auxiliaries
			Modular courses to work in VPH part-time.	1–3 weeks	Official Veterinarians
UK			Course at Bristol or Glasgow Universities No mandatory assessment	2 weeks	Official Auxiliaries
			Practical experience in red and white-meat establishments	7 days	Official Veterinarians

Sources: [12,19].

Differences also exist between Northern Ireland (NI) and the Republic of Ireland (ROI). Following introduction of a MI qualification by the RCVS in the 1950s, ‘Authorised Meat Inspectors’ replaced veterinary-trained inspectors in performing routine MI in NI [19,20]. Conversely in ROI the Department of Agriculture, Food and the Marine (DAFM) employ Temporary Veterinary Inspectors to carry out routine post-mortem MI [21]. However, this system is currently under review: DAFM plan to extend (on a pilot basis) the training of Technical Agricultural Officers to allow them to carry out certain post mortem MI duties (under supervision of the Official Veterinarian) as trained official auxiliaries, on designated species. Their role will change from primarily administrative to one that also includes post-mortem MI (i.e. replacement of Temporary Veterinary Inspectors on the slaughterline) [19,21].

### Non-statutory regulations in Europe

National legislation, codes of practice and membership of Quality Assurance Schemes contribute to variation in MI practices between member states. Codes of practice translate written law into practical guidance for AW standards on farm, during transport and at slaughter with the aim of motivating stockpersons to operate best husbandry practices [22]. Since they are not statutory law, failure of compliance is not an offence in itself.

*Bord Bia* is the Quality Assurance board for producers and processors in ROI: its standards are designed with consideration of the key international and national legislation applicable to AW and pigmeat production [23]. Similarly a number of producers in NI act under the auspices of the United Kingdom’s Red Tractor Farm Assurance Pigs Scheme [24]. Various supermarket chains also have specific standards that their suppliers must adhere to. These may be aligned with or extend minimum legislative requirements depending on the product (e.g. ‘value’ brand vs. free-range) [25].

## Meat inspection data capture and utilisation in EU with special reference to the island of Ireland

### EU regulatory requirements for MI data recording

Though EU Regulation (EC) 854/2004 details conditions to be detected by MI and result in carcass condemnation, there is no legal requirement to employ a standardised recording system (e.g. checklist) [12,26,27].

Regarding utilisation, Regulation (EC) 854/2004 demands significant findings from MI affecting public and animal health to be supplied to the producer and where necessary the private veterinary practitioner responsible for the holding in question [12]. EU law further requires ‘factory returns’ to be sent to producers detailing the following information: carcass number and weight, estimated lean meat content and total price paid [28]. Despite this, Regulation (EC) 854/2004 does not require reasons for or anatomical location of full and partial carcass condemnations to be reported back to producers and veterinarians.

### Variation between EU member states in MI data capture and utilisation practices

Some (EU) member states go beyond EU requirements for MI data capture and utilisation. The Netherlands and Denmark were foremost in developing standardised, computer-based systems for recording and utilisation of pig MI data in the EU [8,29]. Denmark created a national data bank in 1964: the ‘Danish Swine Slaughter Inspection Data System’ [7,30,31]. This was subsequently transformed into a national animal health surveillance scheme in 1978, concurrent with the introduction of an equivalent system in the Netherlands [8,29].

Such initiatives increase the range of pathological conditions recorded during abattoir MI as demonstrated by the recording of atrophic rhinitis that is unique to Scandinavian countries (despite endemic status in most pig-producing countries) [29]. Similarly in the Netherlands MI recording is extended to include offal, skin and limb lesions [26].

A further advantage is that computerised databases facilitate the use of information for surveillance. The Danish pig health scheme aims to identify farms with particularly high prevalences of carcass condemnations and subsequently provide them with the opportunity for expert veterinary assistance [8,31]. This stimulates the development of animal disease prevention strategies at producer level, also promoting improvements in productivity and AW [30,31].

Sweden, Norway, Italy, Luxembourg and Germany are other examples of countries that employ MI databases for animal health surveillance and improvement [19,29,30,32]. The UK has recently introduced similar initiatives for the same reason [33]. In NI the specific cause and anatomical location of pig carcass damage leading to condemnation has been recorded in all abattoirs since 1969 [34,35]. Currently all meat plants in NI use touch screens for data collection; each condition is coded and entered in the screen for carcasses or viscera as appropriate [27]. This is uploaded to APHIS (the Department of Agriculture and Rural Development’s Animal and Public Health Information System) from which it can be accessed by producers and their production advisers (via APHIS–on-Line), and by their private veterinary practitioners (via an internet-based system known as “e-PVP”) [36].

In NI pig MI and carcass quality (e.g. carcass grading, fat class etc.) results are also automatically uploaded from processing plants to an online database through the free Pig Grading Information Scheme (PiGIS), introduced in 2007 [37,38]. Producers registered with the scheme can access real time results of MI for each batch of their animals as regards carcass weight, number of condemnations and quality [37]. Access to records of previous batches and the top producers enables comparison of performance over time and at industry-level for individual producers [38].

Despite these examples of utilisation of MI data in NI, the 2006 FVO inspection of ROI identified areas of non-compliance with EU regulations on recording and communication of inspection results regarding live animals, AW and meat [39]. DAFM responded at the time that a system for feedback of relevant information to managers and associated private veterinary practitioners of the holdings in question was in development [40]. However, in 2008 inadequate feedback from meat plants to producers was reported [41] and linked to a ‘high level of distrust’ for abattoirs by producers in ROI. To date no centralised data capture and utilisation system (as seen in NI) exists in ROI. Recently EFSA [1] highlighted how abattoir data is ‘greatly under-utilised’ and recommended consideration of the potential contribution of MI to pig health and welfare surveillance.

## Potential of meat inspection data as a surveillance tool for (poor) AW

### Evidence of abattoir meat inspection as a surveillance tool for animal health and the potential for extension to AW surveillance

Originally the primary objective of abattoir MI was detection of zoonotic infections. Relatively recently its purpose has been extended by a number of EU member states to encompass surveillance and prevention of animal diseases that pose negligible risk to public health [1,42]. Additionally MI data has been used extensively in epidemiological studies investigating the occurrence of common lesions found at slaughter such as pneumonia, pleurisy, abscessation, ascariasis and tail-biting injuries [5,8,34,43].

In recent years the possibility of extending this approach to encompass AW surveillance has been considered [4,8,18]. Cleveland-Nielsen [7] concluded that abattoir MI might be used as a ‘cheap diagnostic tool’ in herd welfare classification. This is particularly valid in the current context of increasing emphasis on ‘welfare outcomes’ (animal-based measures e.g. lesions from tail-biting) [44]. Though ‘welfare inputs’ have been the focus of AW assessment in the past, it is recognised that such assessments do not guarantee sufficient standards of AW [44-46]. The change in emphasis is reflected by EFSA and the OIE which advocates ‘performance criteria’ as the basis of AW assessment in its guiding principles [18,44]. Additionally the European Commission has funded a Welfare Quality® project (http://www.welfarequality.net) with the purpose of establishing welfare outcome criteria for farm animals, including pigs, at all stages of production. This provides examples of the use of MI data in AW assessment [47].

Since maintenance of good health is the ‘most basic requirement’ for pig welfare [22] the presence of disease and injury at MI may be used to assess AW at farm level [4,8]. Many lesions (e.g. fractures, skin wounds, abscesses) detected at MI may be directly related to suboptimal production systems [2,43,48]. Considerable research on environmental risk factors associated with pulmonary lesions and tail-biting outbreaks in pigs [5,42,43,49-53] has demonstrated that ‘farm level measures’ can decrease the incidence of these diseases [43,48]. Furthermore, Belk et al. [54] discussed how even the prevalence of disease conditions which occur during transport and slaughter (e.g. Porcine Stress Syndrome) may be reduced by changes to on-farm management.

For MI data to be valid for surveillance of AW on-farm a distinction must be made between lesions that occurred as a result of incidents on farm (e.g. tail-biting), during transport or in the lairage of slaughter plants (e.g. acutely fractured limbs). Understanding the relative risks associated with the different stages of the production chain is central to making such distinctions [42,53].

### Limitations of using abattoir MI data as a surveillance tool for AW on-farm

Though using abattoir MI results to measure AW on-farm has many advantages, it is not without limitations. Since slaughter pigs are not representative of national populations there is a risk of bias; fortunately these biases can be assumed to be constant and thus should not interfere with assessment of the prevalence and incidence of conditions over time [1]. Additionally on-farm mortality or euthanasia of pigs, or resolution of lesions that occurred early in production will not be detected by abattoir MI [8]. This will decrease the apparent prevalence of pathologies detected by MI and included in recording databases [1]. However, they are unlikely to negate the overall benefits of such a surveillance scheme; as a continuous process, it offers sustained provision of information regarding animal health and welfare [1].

Another potential limitation is the variation that may exist between abattoirs in the sensitivity and specificity of detection and apparent prevalence of disease conditions observed during MI [27,55,56]. EFSA stressed the variability in sensitivity and specificity of lesion detection at MI; differing time constraints and inspection conditions (e.g. low lighting, crowded pens, pen-side vs. in-movement inspection) could affect the sensitivity of ante-mortem MI [18]. During post-mortem MI the volume and quality of information that can be recorded is limited by throughput, line-speed, intensity of working conditions and recording methods employed [27,29].

The Food Standards Agency in the UK highlights transcription of inspection results to written records as a further source of inconsistency in MI data capture due to variations in the description of identical conditions (e.g. ‘maggots’ vs ‘fly strike’), terminology used at the point of recording (e.g. ‘ascariasis’ vs ‘milk spot’ ) or recording a single cause for condemnation where multiple conditions may exist [27]. The irregularity in terminology and format of additional observations included in inspection reports further contributes to variation in the accuracy and consistency of data captured in MI systems [27].

### Requirements of MI method and data capture for use in surveillance schemes

**Figure 1:**
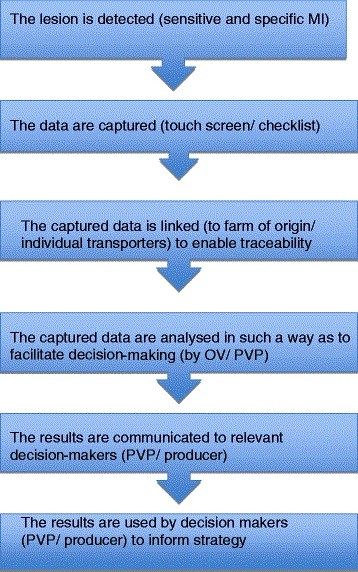
**Key steps for effective utilization of data**[18].

It is critical that MI data capture fulfills a number of requirements before it can be reliably used for surveillance (see Figure 1). Quality of input data is crucial [57,58]. Similarly validation of results is essential to avoid confusion between apparent and true prevalence before application to national databases [55]. Since it is imperative that good quality data are captured [58], workers conducting MI and data capture must be sufficiently trained and motivated to do so as conscientiously as possible [29]. In certain EU member states (including the ROI) the focus of MI training is on protection of public health; as a result this is how MI workers view their role [27]. In NI however there is a longer-standing recognition of the value of post-mortem findings for other purposes, and a significant effort has been made in the past 5 years to ensure consistency of recording and use of terminology at MI across all the slaughter plants in NI. This is facilitated by the centralised unitary nature of NI’s MI service [35].

Additionally, for abattoir surveillance data to be of practical value, relevant information must be made available to decision-makers [1,58]. Currently systematic aggregation of MI data does not occur at EU level, and at a national level is restricted to a selection of countries or regions (e.g. NI, Denmark and the Netherlands) [18].

## Quantification of losses borne from porcine carcass condemnation in Ireland and other EU countries

### Sources of direct economic losses due to poor animal health and welfare in the pork production industry

Quantifying losses at one stage of a chain of interdependent processes is an inaccurate science [59,60]. There are numerous stages in the farm to fork continuum where disease or injury can occur and subsequently result in losses (Table 4). The stage at which rejection of meat or by-products occurs determines which stakeholders must bear such losses.

**Table 4:** Stages at which losses may occur in the pigmeat production chain

**Stage of production**	**Reason for loss**	**Resource in which losses occur**	**Stage loss is incurred**	**Stakeholder incurring losses**
**Farm**	Mortality	Carcass	Farm	Producer
	Clinical Illness	Medicines	Farm	Producer
	Subclinical illness	Carcass	Abattoir	Producer
	Injury	Carcass	Farm	Producer
**Transport/unloading**	Mortality (dead on arrival)	Carcass	Transit	Producer unless very high numbers indicate transporter responsible
	Injuries: fracture/bruise	Carcass	Abattoir	
	Stress	Meat quality	Retail	
	Dehydration	Decreased carcass weight	Abattoir	Producer
**Ante-mortem inspection**	Mortality (death in lairage)	Carcass	Abattoir	Abattoir
	Injuries	Carcass	Abattoir	Abattoir
**Post-mortem inspection**	Disease conditions	Carcass	Abattoir	Producer
	Injuries	Carcass	Abattoir	Producer
	Welfare conditions	Carcass	Abattoir	Producer
	Factory Loss (mangling/contamination)	Carcass	Abattoir	Abattoir
**Carcass grading**	Weight too high/low	Penalty c/kg	Abattoir	Producer
	Poor lean meat %	Penalty c/kg		Producer
**Processor**	Trimmed cuts can’t go for premium products	Decreased value	Retail	Abattoir
**Retailer**	Pale, soft and exudative Dark, firm and dry Trimmed cuts	Decreased retail potential	Retail	Retailer

Sources: [41,54,59,61].

Between the farm and the slaughterhouse there are two main sources of losses to producers: deaths during transport or in abattoir lairage, and partial or entire carcass condemnation at MI [59]. These financial losses to producers can be quantified on the basis of the weight of affected carcasses or carcass parts and the nominal value (per kg) for meat and viscera. However, these are crude estimations since the real value of the meat depends upon carcass weight, anatomical location of rejected parts, grade assigned to the carcass and seasonal price variation [62].

Nevertheless, Hill and Jones employed this approach to their studies on abattoirs in England [63]. In their first study, data were collected during January, March, September and December in one abattoir; a subsample of 168,048 pigs out of the total slaughtered in the abattoir that year (510,880), and extrapolated the results from these four months to estimate annual losses in weight (kg) and value (£). In their second study, seven surveyed abattoirs exhibited variation in region, pig throughput per hour (100–300) and per year (28,000-578,000). In this study, information was collected from the slaughter of 1.3 million pigs in 1980 (9% of total pigs slaughtered in England in 1980). These findings estimated annual losses of over £900,000^1^[62-64]. The estimated financial loss per pig slaughtered ranged to £0.77^1^, a significant part of the profit margin, thus reducing profitability (if not economic viability) of the production stage [63,65]. A study on MI findings in Finland’s largest abattoir also demonstrated significant financial losses associated with the 714,458 pigs slaughtered during 1991[66].

An often underappreciated source of losses to the producer linked to pig welfare on farm is that of growth-retarded pigs. Martinez and others inspected growth-retarded pig carcasses in an abattoir in Spain February-August 2003 and November 2003-March 2004. During this time they found such carcasses had a higher frequency of carcass condemnations than their ‘healthy’ counterparts [67]. From the study population of 6017 pigs with estimated total 2800994 kg slaughtered meat (mean carcass weight: 46.7 kg +/− 15.5), the total condemned weight of meat over the period of study was 24,000kg, equating to direct economic loss to producers of €30,000 (using the market value of pig meat) [67]. Growth retardation also contributes to a wider spread of slaughter weights meaning that producers are more likely to be penalised by processors for supplying carcasses outside the optimal weight bracket [41].

### Sources of indirect losses to industry stakeholders due to poor animal health and welfare in pig production

Disease and welfare problems also incur indirect costs at production level: reduced feed conversion efficiency and increased demand for medicines and labour [22,43,52,67]. Increased prevalence of disease conditions e.g. pleurisy, are also correlated with decreased carcass weight [42,67]. This will always reduce the price paid (per carcass) to the farmer [41].

Processors suffer indirect costs of carcass condemnation through additional inspection of suspect carcasses and disposal of offal or meat unfit for human consumption [68]. Since the efficiency of meat plants is primarily determined by the daily unit output (i.e. number of pigs processed) reducing line speeds for trimming or more detailed inspection processes decreases the efficiency and profitability of the business [69].

Additionally the compromised appearance and quality of affected cuts means they may only be used for lower value products with a lower margin of profit [59]. In France, bruised backfat and hams can be depreciated by 1/3 and 1/5 of their price respectively, whilst in Italy bruised hams are discarded from the high quality Parma ham production line [59]. The particular preferences of retailers as regards lean meat content, backfat thickness and levels of intramuscular fat may also result in penalties to abattoirs and producers who fail to deliver the desired carcass attributes [41].

Losses can also occur due to poor meat quality: pig carcasses are graded based on P2 backfat (i.e. lean meat content); this determines their value [41]. Despite being fit for human consumption, Pale, Soft and Exudative (PSE) and Dark, Firm and Dry (DFD) meat has reduced retail potential and profitability due to its abnormal texture, appearance and odour [70-72]. Such meat quality defects have cost the UK pigmeat industry an estimated £12,660,000 annually [73].

### Current role of the industry in promoting the use of MI data to improve animal health and welfare

At present utilisation of MI data as an AW (or even as an animal health) surveillance tool at European level is inadequate [18]. This is in spite of its extensive usage in epidemiological studies on animal health and welfare [34,67,74] and surveillance schemes run by individual EU Member states [30,31].

However, in response to a study by BPEX [75], which estimated losses on the scale of c.€130 million per week to the pig industry in Europe, industry-led initiatives are attempting to reduce losses throughout pig production, particularly those associated with carcass condemnation [8]. For example *‘Farmingnet’* is currently employed in the Netherlands, UK and Germany. It allows producers to access slaughter information about each batch they send to a certain meat company regarding average and standard deviation of weight and probe, price deviation €/kg from the maximum possible and batch health information [58].

Scotland created the voluntary *Wholesome Pig Scheme* in 2003, collecting MI data for animal health surveillance based on Danish and Dutch systems [8]. BPEX introduced an identical, levy-based scheme in England and Wales in 2005 [33]. Having subscribed producers receive information sheets which provide a score based on prevalence and severity of conditions found in their batch of animals, as well as an explanation of associated risk factors and preventative measures [33]. The objectives are to estimate the prevalence and extent of carcass condemnation in the pig herd in Great Britain whilst encouraging the utilisation of MI data by producers [33].

Similarly NI introduced the Pig Grading Information System (PiGIS) which enables producers to gain information from MI regarding grading, weight, condemnations and carcass quality of their livestock [38]. Additionally PiGIS aims to motivate producers to make improvements by providing access to the same results from ‘top’ producers [38]. In ROI there is currently no such national or continuous surveillance scheme in operation.

## Discussion

Across the EU there is significant variation in implementation of EU legislation regulating MI. FVO reports demonstrate minor areas of non-compliance in almost all EU member states, including ROI and UK (the latter of which NI is assessed as a component) [3,39]. However, much of the variation that exists is borne from areas or member states that are compliant, suggesting that a weakness in EU MI legislation is primarily responsible for such variation. Training and employment requirements are also likely to affect the sensitivity and specificity of detection of pathological conditions during MI. This may result in increased or decreased condemnations, different classifications of local or generalised conditions and thus affect the apparent prevalence of carcass condemnations and diseases in different member states.

Considerable variation between member states also exists in data capture and utilisation, due to a laxity of EU legislation in these areas. Denmark and the Netherlands set a high standard with respect to standardisation, recording, communication and storage of MI data, enabling its effective utilisation by various industry stakeholders for surveillance and improvement of public and animal health, as does NI, although its uptake and use for these purposes is perhaps not as advanced. In the absence of such stringent standards across the EU, meat companies and industry bodies in a number of member states have developed various initiatives modeled on the Dutch and Danish systems [8]. The need for such initiatives is long overdue; MI is recognised as a valuable tool in continuous assessment of the prevalence of animal diseases [76] as well as ‘ideal for monitoring lesion incidence and severity’ in cases of subclinical (pulmonary) lesions [5]. Unfortunately some member states, including ROI, still have no such scheme operating on a regular basis or a scale greater than individual meat companies. In these cases reporting of many lesions affecting animal health and welfare is done on a purely ad-hoc basis reliant on the conscientiousness of individual workers. In design of such systems it is imperative that their implementation is feasible in current systems [56].

In the current environment of AW assessment which emphasises the use of ‘welfare outcomes’ rather than ‘welfare inputs’, MI presents itself as a potential surveillance tool for AW both on- and post-farm [4]. Indeed ante- and post-mortem MI stages are incorporated in the *Welfare Quality***®** assessment stages [47]. Further, EFSA’s proposed changes to MI strive to incorporate assessment of meat quality aspects [1] such as carcass bruising, which reflect poor AW, increasing the potential usefulness of MI as a welfare surveillance tool.

The main issues hindering wide scale adoption of MI as an AW surveillance tool are cost, logistics and inconsistencies in MI method and recording between and within member states and abattoirs [56].

Losses associated with poor health and welfare are substantial and extend the length of the pigmeat production chain. Their quantification demonstrates to producers that poor husbandry practices and environment on-farm can result in economic losses, reversing the putative relationship between increased farm AW and increased production costs. In a market-driven industry with a small margin of profit and no stabilization by EU or national subsidies, such as pigmeat production, such information has the power to motivate farmers to improve production efficiency by reducing disease and welfare issues in their herds.

## Conclusion

Carcass condemnation of pigs at MI is a substantial source of direct and indirect economic losses to producers, processors and other industry stakeholders. Consequently, improved animal health and welfare in production systems has the potential to reduce inefficiencies in the pig industry.

**Figure 2:**
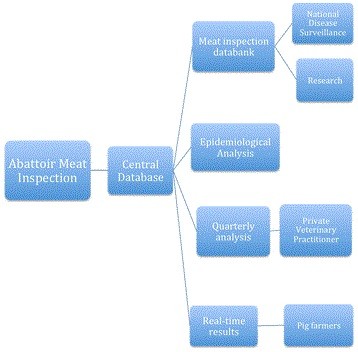
**Diagram representing the flow of information in the UK pig health schemes, starting with the collection of electronic records of the gross pathology results in the abattoirs, to the summary reports sent to the producers and veterinary advisers.** Modified from [29].

Abattoir MI data is a valuable tool for animal health surveillance, as exhibited by its extensive usage in national databases and epidemiological studies. MI data should be extended to facilitate surveillance of AW within and between EU member states. The proposed changes to MI to include a focus on meat quality aspects would enable welfare issues to be included in such surveillance processes. Additionally increased feedback of MI results to producers will promote its use in herd health programmes and subsequent improvements in animal health, AW and productivity (see Figure 2).

## Abbreviations

MI, Meat Inspection; AW, Animal Welfare; ROI, Republic of Ireland; NI, Northern Ireland; EU, European Union; EFSA, European Food Safety Authority; DAFM, Department for Agriculture Food and the Marine; DARD, Department for Agriculture and Rural Development; DEFRA, Department for Environment Food and Rural Affairs; FAO, Food and Agriculture Organization of the United Nations; OIE, World Organization for Animal Health; FSAI, The Food Safety Authority of Ireland; FAWAC, The Farm Animal Welfare Advisory Council; RCVS, Royal College of Veterinary Surgeons.

## Competing interests

There were no competing interests between the authors and other organisations or individuals that would have affected the outcome of the review.

## Authors’ contributions

SH wrote the manuscript. SM and AH directed the structure and content of the paper. All authors provided advice to the lead author. All authors participated in reviewing the manuscript, and read and approved the final manuscript.
